# Clinical utility of methicillin-resistant *Staphylococcus aureus* nasal PCR to streamline antimicrobial use in treatment of diabetic foot infection with or without osteomyelitis

**DOI:** 10.1186/s12879-023-08248-2

**Published:** 2023-05-05

**Authors:** Gaielle Harb, Teri Hopkins, Linda Yang, Kathleen Morneau, Jose Cadena-Zuluaga, Elizabeth Walter, Christopher Frei

**Affiliations:** 1 South Texas Veterans Health Care System, San Antonio, U.S.; 2 Long School of Medicine, University of Texas Health Science Center at San Antonio, San Antonio, U.S.; 3 College of Pharmacy, University of Texas at Austin, Austin, U.S.

**Keywords:** Methicillin-resistant *Staphylococcus aureus*, Polymerase chain reaction, Diabetic foot, Antibacterial agents, Drug utilization

## Abstract

**Background:**

Diabetic Foot Infection (DFI) guidelines recommend empiric methicillin-resistant *Staphylococcus aureus* (MRSA)-targeted therapy in settings where there is high prevalence of MRSA infections or in cases of severe infection; however, they do not provide recommendations for de-escalation. This approach has the potential to increase unnecessary use of broad-spectrum antibiotics; therefore, additional strategies are needed to optimize appropriate antibiotic use. This study evaluates the effect of MRSA nasal PCR testing on MRSA-targeted antibiotic use and clinical outcomes in patients with DFI.

**Methods:**

This was a retrospective quasi-experimental study of patients admitted to South Texas Veterans Health Care System for DFI, with or without osteomyelitis (OM), who had an MRSA nasal PCR and culture data. Eligible patients were identified from the Corporate Data Warehouse and reviewed via electronic health record. Patients were allocated into two groups: PRE (5/1/2019-4/30/2020) and POST (12/1/2020-11/30/2021) protocol implementation for de-escalation or avoidance of MRSA-targeted antibiotics. The primary outcome was median (interquartile range [IQR]) hours of empiric inpatient MRSA-targeted antibiotic therapy. A Wilcoxon Rank Sum test was used to assess the difference between the groups for the primary outcome. Secondary outcomes included the proportion of patients needing MRSA coverage added back for MRSA after de-escalation, hospital readmission, length of hospital stay (LOS), patient mortality, and acute kidney injury.

**Results:**

A total of 151 patients were included (83 PRE; 68 POST). Most patients were male (98% PRE; 97% POST) with a median age of 64 (IQR, 56–72) years. Incidence of MRSA in DFI in the cohort was 14.7% overall (12% PRE and 17.6% POST). MRSA was detected via nasal PCR in 12% of patients 15.7% PRE and 7.4% POST). After protocol implementation, there was a significant decrease in empiric MRSA-targeted antibiotic therapy use, from a median of 72 (IQR, 27–120) hours in the PRE group, to 24 (IQR, 12–72) hours in the POST group (p < 0.01). No significant differences were found for other secondary outcomes.

**Conclusion:**

This study of patients presenting to a Veterans Affairs (VA) hospital with DFI identified a statistically significant decrease in median duration of MRSA-targeted antibiotic use post-protocol implementation. This suggests a favorable effect of MRSA nasal PCR for de-escalation or avoidance of MRSA-targeted antibiotics in DFI.

**Supplementary Information:**

The online version contains supplementary material available at 10.1186/s12879-023-08248-2.

## Background

Inappropriate use of broad spectrum antibiotics is an ongoing global health emergency with serious consequences, which include multidrug-resistant organisms (MDROs), longer LOS, adverse drug events, and risk of *Clostridioides difficile* [1, 2]. Current Infectious Diseases Society of America (IDSA) DFI guidelines recommend empiric MRSA-targeted therapy when there is high local prevalence of methicillin-resistant *Staphylococcus aureus* (MRSA) infections or in cases of severe infection; however, they do not make recommendations on de-escalation. [3]. Though these guidelines have been archived by IDSA, clinicians are still practicing based on these recommendations. Additionally, the most recent guideline by the International Working Group on the Diabetic Foot recommend empiric MRSA-targeted therapy in patients with MRSA risk factors; however, they do not define these risk factors or high [4]. This highlights the need for more updated data. These recommendations may increase unnecessary use of broad-spectrum antibiotics, leading to adverse events. This has prompted investigation into strategies to optimize antibiotic use in this patient population.

One emerging strategy includes the utilization of MRSA nasal [5]. This is a nasal swab test that is used to identify MRSA colonization. Patients without MRSA colonization are at low risk for having MRSA as a causative organism, allowing for quick de-escalation from empiric MRSA-targeted therapy. The MRSA nasal PCR has been widely utilized for MRSA-targeted therapy de-escalation in pneumonia, given negative predictive values (NPV) have been > 90% in various studies [6–8]. Data suggest pharmacist-ordered MRSA nares testing reduces duration of empirical anti-MRSA therapy and rates of AKI in patients with suspected pneumonia with no effect on clinical cure [9]. DFIs are one of the most common infections treated in the hospital and these infections often result in prolonged courses of antibiotics. Consideration for adding MRSA coverage in DFI is appropriate in our patient population since MRSA prevalence is > 30% locally. Furthermore, there is growing literature to suggest MRSA nasal PCR also carries high NPV in DFI, with one study showing a NPV of 94.4% [10, 11]. However, limited knowledge exists on how utilization of MRSA nasal PCR can influence antimicrobial use in DFI.

In response to internal data demonstrating a 94% NPV for MRSA nasal PCR in DFI, our institution implemented an intervention to utilize this test for early de-escalation or avoidance of empiric MRSA-targeted antibiotics in [12]. In December 2020, a local clinical pathway for DFI treatment and electronic order menus were updated to include a MRSA nares lab order within the vancomycin order set. This was done to guide de-escalation of MRSA coverage in the setting of a negative MRSA nasal swab in patients without cultures growing MRSA from DFI cultures in the past year. Education was also provided to medicine attendings and clinical pharmacy specialists. This study aims to evaluate the impact of this intervention. To our knowledge, this is the first study to evaluate the effect of MRSA nasal PCR testing on MRSA-targeted antimicrobial use and clinical outcomes in patients with DFI with or without OM at admission.

## Methods

### Study design

This was a retrospective quasi-experimental study of patients admitted with a DFI diagnosis, with or without OM, to the South Texas Veterans Health Care System. The University of Texas Health at San Antonio Institutional Review Board (San Antonio, TX, USA) conducted a review through the Office of Clinical Research. The study was approved under non-regulated research, was exempt from Institutional Review Board review, and classified as quality improvement. It was a retrospective chart review of an intervention that had already been made under our local antimicrobial stewardship program as quality improvement, as such informed consent was waived by the University of Texas Health at San Antonio Institutional Review Board (San Antonio, TX, USA) because this intervention was not made under a research protocol. All methods were performed in accordance with the institution relevant guidelines and regulations.

### Study subjects

Patients with DFI were identified using ICD10 codes and confirmed via chart review. Patients were included if they were ≥ 18 years and had an MRSA nasal PCR with culture data obtained from site of diabetic foot infection during the same admission. Culture data included swab, wound, tissue, abscess, and bone cultures. Patients were excluded if they had history of MRSA infection within 1 year prior to the index admission for DFI. This exclusion criterion was necessary because previous infection with MRSA in the past 12 months is an independent risk factor for MRSA DFI. Additionally, these patients may have received antibiotics targeting MRSA which could result in nasal decolonization. Patients were allocated into two groups: PRE (5/1/2019-4/30/2020) and POST (12/1/2020-11/30/2021) protocol implementation for de-escalation or avoidance of MRSA-targeted antibiotics. Repeat patients were included in the study, as long as they had not isolated MRSA within the past year, as this was a separate exclusion criterion.

A 7-month wash-out period was allotted between each study group to allow for provider education after protocol implementation. This washout period was determined to have a clear delineation of the time periods before and after the intervention was implemented and complete acceptance of the protocol that was the basis for the intervention.

The protocol was approved by our local Antibiotic Subcommittee and P&T, and introduced to our internal medicine attendings during one of their monthly meetings. The recommendations were also implemented into electronic order menus in the electronic medical record. Clinical pharmacy specialists on inpatient medicine teams also assisted with carrying out the intervention.

### Data collection

Data were collected from the Veterans Health Administration’s (VHA) Corporate Data Warehouse (CDW) and retrospective chart review.

### Statistical analysis

Descriptive data for the entire study population were defined as percentages for nominal data and as median and interquartile range (IQR) for numerical data. The primary outcome was median [IQR] hours of empiric inpatient MRSA-targeted antibiotic therapy. Empiric antibiotics were defined as MRSA-targeted antibiotic therapy if the antibiotic of choice had activity against MRSA. For this study, these included vancomycin, daptomycin, linezolid, ceftaroline, dalbavancin, minocycline, and doxycycline. Duration of empiric inpatient MRSA-targeted antibiotic therapy collected using pharmacy data in the form of medication administration records. A Wilcoxon Rank Sum test was used to assess the difference between the groups for the primary outcome. A sample size of 32 patients in total was estimated to meet 80% power for the primary outcome. Secondary outcomes included the proportion of patients needing MRSA coverage added back for MRSA after de-escalation, 9-month hospital readmission due to DFI, LOS, in-hospital mortality, and AKI. We defined AKI as an increase of serum creatinine by at least 0.3 mg/dL over a 48-hour period from the baseline serum creatinine at admission. For numerical secondary endpoints, Wilcoxon Rank Sum test was used to assess the difference between the groups. For categorical secondary endpoints, a chi-square or Fisher’s Exact test was used to assess the difference between the groups. Multivariable standard least squares regression (for duration) and nominal logistic regression (for hospital 9-month readmission due to DFI) were constructed with PRE/POST as the independent variable, outcome as the dependent variable, and divergent baseline characteristics (p < 0.05) as covariates. All statistical analyses were performed with JMP Pro 14 ® (SAS Institute, Cary, NC).

## Results

**Fig. 1:**
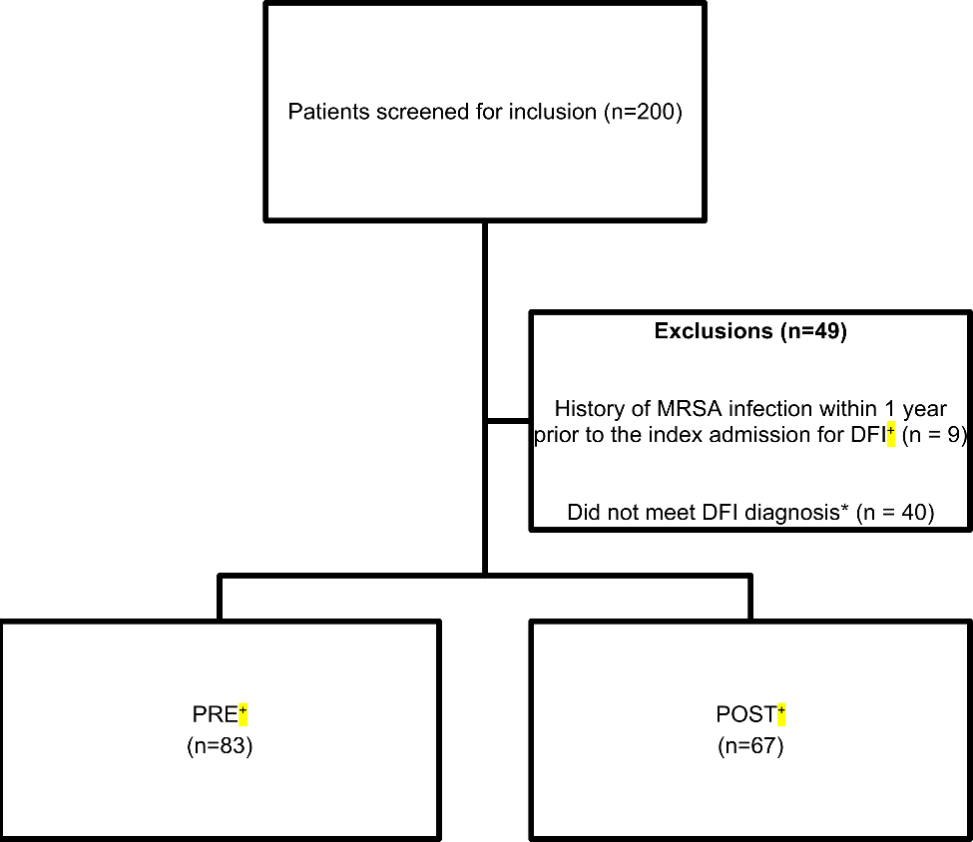
Patient Selection ^+^Abbreviations: methicillin-resistant *Staphylococcus aureus* (MRSA), diabetic foot infection (DFI), pre-protocol implementation (PRE), post-protocol implementation (POST). *Patients with ICD9 or ICD10 codes for DFI, but had no provider documentation, provider intervention, or clinical data to suggest DFI was a listed problem during their hospital admission.

Two hundred patients were screened for inclusion; 151 met study criteria and were included in the analysis. The primary reasons for exclusion were history of MRSA infection within 1 year prior to the index admission for DFI and/or not having a DFI diagnosis during the chart review (verification step) (Fig. 1). PRE and POST groups were similar with regard to sex at birth (98% PRE; 97% POST; p = 1.00) and median age (67 years PRE; 64 years POST; p = 0.11).Five characteristics were significantly different at baseline: malignancy (p = 0.02), swab culture (0.03), abscess culture (0.04), MSSA (0.03), Gram-negative (0.02). (Table 1).

**Table 1:** Baseline Characteristics

	PRE (n = 83)	POST (n = 68)	P-value
Age (median [IQR])	66.8 [56.1–72.9]	63.7 [55.7–68.5]	0.11
Male (%)	97.6	97.1	1.00
A1C^+^ (mean)	8.3 ± 2.2	8.5 ± 1.9	0.63
Comorbid conditions (%) Peripheral vascular disease Transplant Malignancy OM	27 (32.5) 1 (1.2) 11 (13.2) 36 (43.3)	22 (32.4) 1 (1.5) 2 (2.9) 36 (52.9)	0.98 1.00 0.02 0.24
Microbiologic cultures (%) Swab Wound Tissue Abscess Bone	6 (7.2) 33 (39.7) 42 (50.5) 16 (19.2) 46 (55)	0 26 (38.2) 44 (64.7) 5 (7.4) 30 (44.1)	0.03 0.85 0.08 0.04 0.17
Organisms isolated (%) MRSA^#^ MSSA^*^ Other Gram positive Gram negative Anaerobes Culture negative	10 (12.0) 18 (21.7) 41 (49.3) 38 (45.8) 14 (16.9) 13 (15.7)	12 (17.6) 26 (38.2) 36 (52.9) 18 (26.4) 8 (11.8) 9 (13.2)	0.34 0.03 0.66 0.02 0.38 0.67

^+^The most recent A1C value was obtained at the time of hospitalization.

^#^Methicillin resistant *Staphylococcus aureus*

*Methicillin susceptible *Staphylococcus aureus*

MRSA was detected via nasal PCR in 12% of patients (15.7% PRE and 7.4% POST). The NPV was found to be 94% PRE and 95% POST. There was a significant decrease in empiric MRSA-targeted antibiotic therapy use, from a median of 72 (IQR, 27–120) hours in the PRE group to 24 (IQR, 12–72) hours in the POST group (p < 0.01) (Table 2). In a multivariable standard least squares regression model with PRE/POST as the independent variable, duration of empiric MRSA-targeted antibiotic therapy as the dependent variable, and the five divergent baseline characteristics as covariates, the intervention remained significant for a shorter duration of empiric MRSA-targeted antibiotic therapy use in the POST period (p < 0.01).

**Table 2:** Outcomes

	PRE (n = 83)	POST (n = 68)	P-value
Primary Outcome			
Duration of empiric MRSA-targeted antibiotic therapy (hours, median [IQR])	72 [27–120]	24 [12–72]	< 0.01
**Secondary Outcomes**			
MRSA coverage added back for MRSA (%)	0.0	0.0	---
AKI (%)	15.7	6.1	0.07
LOS (days, median [IQR])	8.0 (5.0–13.0)	9.0 (6.3–14.0)	0.32
In-hospital mortality (%)	2.4	2.9	1.00
9-month readmission due to DFI (%)	18.1	31.3	0.06

Abbreviations: methicillin-resistant *Staphylococcus aureus* (MRSA), acute kidney injury (AKI), length of stay (LOS), interquartile range (IQR), pre-protocol implementation (PRE), post-protocol implementation (POST)

No significant differences were found for secondary outcomes (Table 2); however, the rate of AKI in the PRE group (15.7%) was numerically higher than the rate of AKI in the POST group (6.1%) (p = 0.07). No patients in either group required MRSA coverage added back for MRSA after initial de-escalation. Additionally, the median LOS was 8 days in the PRE group and 9 days in the POST group (p = 0.32). All-cause hospital mortality was 2.4% in the PRE group and 2.9% in the POST group (p = 1.00). Lastly, the 9-month hospital readmission due to DFI rate was 18.1% in the PRE group to 31.3% in the POST group (p = 0.06). A multivariable nominal logistic regression model was also performed with PRE/POST as the independent variable, hospital readmission as the dependent variable, and the five divergent baseline characteristics as covariates. PRE/POST group was not significantly predictive of hospital readmission in the multivariable regression model.

We also collected 6-month, 9-month, and 12-month all cause hospital readmission as well as 6-month, 9-month, and 12-month hospital readmission due to DFI, though 10 patients in the POST group had missing 12-month follow-up data due to the study period (Table 3). Given the numerically higher rates of 9-month readmission in the POST-intervention group, we evaluated these cases individually to determine if withholding vancomycin was likely to contribute to this outcome. Of the 21 patients readmitted, there were only four cases with pathogens that might have required vancomycin, and only one patient that seemed to be missing coverage after further review; that organism was *Corynebacterium striatum*, which did grow in bone, but has questionable pathogenic potential in a polymicrobial culture.

**Table 3:** Hospital Readmissions

	6-month	9-month	12-month
PRE (n = 83)			
Hospitalization (all cause), n (%)	35 (42.2)	35 (42.2)	37 (44.6)
Hospitalization (due to DFI^+^), n (%)	14 (16.8)	15 (18.1)	16 (19.3)
**POST (n = 68)**			
Hospitalization (all cause), n (%)	31 (45.6)	34 (50)	36 (52.9)^*^
Hospitalization (due to DFI), n (%)	19 (27.9)	21(30.9)	22 (32.4)^*^

^+^Diabetic foot infection

*Ten patients in the post group did not have information available for the 12-month timepoint

## Discussion

This single-center, retrospective, quasi-experimental study assessed the effect of MRSA nasal PCR testing on MRSA-targeted antimicrobial use and clinical outcomes in patients with DFI with or without OM. Although the current IDSA DFI guidelines only recommend empiric MRSA-targeted therapy when there is high local prevalence or in cases of severe infection, there is no recommendation for de-escalation, which may lead to an overutilization of anti-MRSA [2]. Based on previous internal data, we chose to primarily assess what effect MRSA nasal PCR would have on anti-MRSA antimicrobial use at our institution along with other safety and clinical [12].

We found a significant difference between the groups for duration of empiric MRSA-targeted therapy. It is important to note that no patients needed MRSA coverage added back due to isolation of MRSA in clinical cultures after initial anti-MRSA therapy discontinuation. This suggests that the use of MRSA nasal PCR as a de-escalation tool not only decreased antibiotic hours, but also did not directly worsen clinical outcomes. Additionally, from a safety standpoint, there was a trend towards decreased rates of AKI in the POST group, which may increase morbidity and cost. There was also a numerical difference in readmission rates with a trend towards higher rates of 9-month readmission due to DFI in the POST group. This could be due to differences in source control in the two arms that was not assessed. In our review of the readmissions in the POST group, all but one of the readmissions did not appear to be related to withholding vancomycin therapy.

Our findings are consistent with published data to support the use of MRSA nasal PCR as a de-escalation tool, which first emerged in patients with pneumonia. Baby and colleagues performed a retrospective analysis of patients who received either vancomycin or linezolid for suspected pneumonia before and after the implementation of a pharmacist-driven protocol for MRSA nasal PCR [9]. They found that the use of MRSA nasal PCR testing in patients with suspected MRSA pneumonia reduced the duration of empiric MRSA-targeted therapy by approximately 2 days without increasing adverse clinical outcomes such as days to clinical improvement, length of hospital stay, or hospital mortality. A growing number of studies have been done to assess utility of MRSA nasal PCR in patients with DFI; however, this is the first study that we know of to report the effect on antimicrobial use in patients with [10–12]. We examined the utility of using an MRSA nasal PCR testing protocol as a de-escalation tool in DFI. Our primary outcome of reduced antibiotic hours aligns well with the previously published study for pneumonia.

Our study has limitations. No blinding was conducted as part of the chart review. The single-center design and veteran population limit generalizability to other populations and institutions. Given the culture specimens are labeled by the surgeons or other providers who obtained them, we are unable to verify whether they are correctly labeled, and not all cultures were obtained surgically. There are significant differences in percentage of MSSA and Gram-negative pathogens isolated in cultures. Our facility did see an increase in prevalence of MSSA between the PRE and POST time periods; however, we are unable to determine a reason for the difference in Gram negatives. We did not assess antibiotics administered in the emergency department or prescribed at discharge, so the reported empiric MRSA-targeted antibiotic therapy use in hours may be underestimated given the duration of treatment for DFI with OM. Additionally, although the aim of this study was not to assess NPV of MRSA nasal PCR, our findings are consistent with other studies in patients with DFI. We did not evaluate patients if they had history of MRSA infection within 1 year prior to the index admission for DFI, which limits our ability to utilize MRSA nasal PCR as a de-escalation tool in this patient population. We also did not collect information on surgical margins given our study includes both DFI with or without OM. We also only treated AKI as a binary variable and did not assess the long-term impact of the therapies on kidney function. Furthermore, we only reported if MRSA coverage was added back for MRSA, which decreases generalizability as vancomycin is not only used for MRSA, but also for beta-lactam resistant organisms, such as *Corynebacterium striatum* or coagulase negative staphylococci. However, these organisms are often indolent and the risk of waiting for cultures to finalize before initiating therapy is low. We also did not account for variables such as source control, outpatient antibiotic selection, and medication adherence which could have affected hospital readmission rates.

Hospital readmission alone is not the most important or relevant outcome, and it is driven by many factors (e.g., source control, social determinants of health, etc.). Taken together, we believe our secondary outcomes are necessary to get a more complete picture of the patients’ experience. Collectively, we believe the chosen primary and secondary outcomes make the case that treatment outcomes of MRSA DFI were similar in the pre- and post-intervention periods.

Though we conducted a sample size calculation for the primary outcome (median hours of inpatient MRSA-targeted antibiotic therapy) before beginning our study, we did not conduct a sample size calculation for the secondary outcomes, including hospital readmission, and it is possible that we did not have enough patients to eliminate the chance of type 2 error for our secondary outcomes. Table 3 depicts the raw numbers for the secondary outcomes involving hospital readmission, but health outcomes such as these are multifactorial, and this study was not designed to collect all the factors that might have had an impact on the secondary outcomes (e.g., source control, social determinants of health, etc.). Furthermore, we did not have 12 months of follow-up for all patients in the post-intervention group. Finally, the VA cost for labs and medications is proprietary, so we cannot determine or report the cost-benefit of the intervention. Nevertheless, we believe this intervention is valuable as an antimicrobial stewardship initiative.

## Conclusion

Based on the finding of our study, MRSA nasal PCR should be utilized as a de-escalation tool for patients admitted to our facility for DFI with or without OM who do not have a history of MRSA infection within the prior year. Larger prospective studies are needed to demonstrate additional benefits. This study of patients presenting to a VA hospital with DFI identified a statistically significant decrease in median duration of inpatient MRSA-targeted antibiotic use post-protocol implementation. This suggests a favorable effect of MRSA nasal PCR for de-escalation or avoidance of MRSA-targeted antibiotics in DFI.

## Electronic supplementary material

Below is the link to the electronic supplementary material.

Supplementary Material 1

## Data Availability

The datasets used and/or analyzed during the current study are available from the corresponding author on reasonable request.

## List of abbreviations

AKI: cute kidney injury
CDW: corporate data warehouse
DFI: diabetic foot infection
IQR: interquartile range
IRB: institutional review board
LOS: hospital length of stay
MDROs: multidrug-resistant organisms
MRSA: methicillin-resistant *Staphylococcus aureus*
MSSA: methicillin-susceptible *Staphylococcus aureus*
NPV: negative predicative value
OM: osteomyelitis of the foot
VHA: Veterans Health Administration
