# Supplementary material for: Clinical utility of methicillin-resistant Staphylococcus aureus nasal PCR to streamline antimicrobial use in treatment of diabetic foot infection with or without osteomyelitis
Source: BMC Infect Dis. 2023 May 5;23:297. doi: 10.1186/s12879-023-08248-2 (PMC10163799; doi:10.1186/s12879-023-08248-2)
Supplement: Supplementary file 1 — Supplementary Material 1 [file 12879_2023_8248_MOESM1_ESM.docx]

**Appendix 1.** ICD 9 and 10 codes

| **Comorbidity** | **ICD 9 or 10 Code** |
| --- | --- |
| Diabetic foot infection | E08; E09; E10; E11; E12; E13 |
| Peripheral vascular disease | I73.8; I73.9 |
| Solid organ transplant | Z94 |
| Malignancy | C17.0; C17.1; C17.2; C17.3; C17.8; C18.0; C18.1; C18.2; C18.3; C18.4; C18.5; C18.6; C18.7; C18.8; C21.1; C21.2; C21.8; D12.0; D12.1; D12.2; D12.3; D12.4; D12.5; D12.6; D12.7; D12.8; C56.1; C56.2; C57.01; C57.02; C48.0; C48.1; C48.8; Z85.03; C54.0; C54.1; C54.2; C54.3; C54.8; D07.0; D25.0; D25.1; D25.2; C25.0; C25.1; ;C25.2; C25.3; C25.4; C25.5; C25.6; C25.7; C25.8; D13.6; D13.7; C16.0; C16.1; C16.2; C16.3; C16.4; C16.8; D13.1; Z85.020; C64.1; C64.2; C65.1; C65.2; C66.1; C66.2; C67.0; C67.1; C67.2; C67.3; C67.4; C67.5; C67.6; C67.7; C67.8; C68.0; C68.1; C68.8; Z85.520; Z85.528; Z85.53; Z85.54; Z85.51; Z85.59; C43.0; C43.11; C43.12; C43.21; C43.22; C43.31; C43.39; C43.51; C43.52; C43.59; C43.61; C43.62; C43.71; C43.72; Z85.820; Z85.828; Z85.850; Z85.858; C73; C74.01; C74.02; C75.0; C75.1; C75.2; C75.3; C75.4; C75.5; C69.22; C69.31; C69.32; C69.41; C69.42; C70.0; C70.1; C71.0; C71.1; C71.2; C71.3; C71.4; C71.5; C71.6; C71.7; C71.8; Z85.840; Z85.841; Z85.848; C80; C80.1 |
| Osteomyelitis | M86.0; M86.1; M86.2; M86.3; M86.4; M86.5; M86.6; M86.7; M86.8; M86.9 |
